# Peptide-Based siRNA Nanocomplexes Targeting Hepatic Stellate Cells

**DOI:** 10.3390/biom13030448

**Published:** 2023-02-28

**Authors:** Chien-Yu Lin, Umar-Farouk Mamani, Yuhan Guo, Yanli Liu, Kun Cheng

**Affiliations:** Division of Pharmacology and Pharmaceutical Sciences, School of Pharmacy, University of Missouri-Kansas City, 2464 Charlotte Street, Kansas City, MO 64108, USA

**Keywords:** liver fibrosis, siRNA, nanocomplex, IGF2R, *PCBP2*, glutamate

## Abstract

Liver fibrosis is the excessive accumulation of extracellular matrix (ECM) in the liver due to chronic injuries and inflammation. These injuries activate and transform quiescent hepatic stellate cells (HSCs) into proliferative myofibroblast-like cells, which are the key contributors to the secretin of ECM in the fibrotic liver. The insulin-like growth factor 2 receptor (IGF2R) is a multifunctional receptor that is overexpressed on activated HSCs and is a specific molecular marker of activated HSCs in the fibrotic liver. We recently discovered an IGF2R-specific peptide that significantly increases the binding affinity and uptake of a protein-based siRNA nanocomplex to activated HSCs. However, there is a potential concern about the immunogenicity of protein-based siRNA delivery systems. In this study, we used the IGF2R-specific peptide to modify a small peptide-based siRNA nanocomplex for HSC-specific drug delivery. We incorporated a short spacer and glutamate residues into the IGF2R peptides. The siRNA nanocomplex modified with the IGF2R-3GK6E peptide demonstrated higher HSC specificity compared to an unmodified nanocomplex. This peptide-based nanocomplex provides a promising platform to effectively deliver *Pcbp2* siRNA to activated HSCs for the treatment of liver fibrosis.

## 1. Introduction

Liver fibrosis is characterized by the excessive accumulation of extracellular matrix (ECM) in the liver due to chronic inflammation and injuries, such as viral hepatitis, alcohol abuse, nonalcoholic steatohepatitis, and toxins [1,2]. Chronic injury and the uncontrolled accumulation of ECM alters the tissue structure of the liver and leads to the development of fibrosis, which can progress to irreversible cirrhosis and hepatocellular carcinoma (HCC) if left untreated [3,4]. Hepatic stellate cells (HSCs) represent 5–8% of liver cells, but they play a critical role in the development of liver fibrosis [5]. When the liver is injured, quiescent HSCs are activated by pro-fibrotic mediators and transformed into proliferative myofibroblast-like cells, which secrete ECM and contribute to the development of fibrosis [4,6]. Several strategies have been developed to target HSCs in order to reduce fibrosis, such as inhibiting their activation and proliferation of activated HSCs, suppressing their pro-inflammatory responses, inducing their apoptosis, and reducing their ability to stimulate angiogenesis [7].

Alpha-complex protein-2 (αCP2), an RNA binding protein encoded by the poly(rC) binding protein-2 gene (*PCBP2*), is overexpressed by activated HSCs during liver fibrogenesis. It binds to the C-rich region of the 3′ untranslated region (UTR) of collagen α1(I) mRNA, stabilizing and increasing the half-life of the mRNA, and leading to the accumulation of type I collagen in the fibrotic liver [8,9]. Small interfering RNAs (siRNAs), which are 21–23 base pairs in length, are a useful tool for specifically silencing target genes. Once inside cells, the double-stranded siRNA is incorporated into the RNA-induced silencing complex (RISC), which then unwinds to form a single-stranded siRNA. The functional antisense strand of the siRNA guides the RISC to recognize its complementary mRNA and to induce the degradation of target mRNAs [10]. In our previous studies, siRNA targeting the *Pcbp2* gene was found to effectively reduce the levels of the *Pcbp2* mRNA and αCP2 protein, and to reverse the overexpression of type I collagen in activated HSCs exposed to alcohol and profibrotic cytokine [11]. Silencing the *PCBP2* gene with siRNAs is a potential antifibrotic therapy for the fibrotic liver. However, due to the low delivery efficiency, rapid enzymatic degradation, and high renal clearance rate of free siRNAs, it is challenging to deliver free siRNAs to the target sites. A nanoparticle is a promising platform to overcome these barriers and reduce undesirable side effects of siRNAs [12]. Recently, we developed targeted neutravidin-based nanocomplexes that can specifically deliver siRNAs to activated HSCs [13,14]. These nanocomplexes were effective in targeting and reversing carbon tetrachloride (CCl_4_)-induced liver fibrosis, however, the immunogenicity of protein-based therapeutics is still a potential concern [15]. Therefore, it is essential to develop a safe and efficient delivery system for siRNA. We have developed a peptide-based siRNA delivery system that can self-assemble into a micelle-like structure and effectively condense with siRNAs [16]. The cholesteryl peptides/siRNA nanocomplex has been shown to enhance the transfection efficiency of siRNA into cancer cells and fibroblasts and to effectively regulate gene silencing activity [16,17,18].

Several biomarkers that are overexpressed in activated HSCs—such as retinol-binding protein (RBPR), insulin growth factor 2 receptor (IGF2R), platelet-derived growth factor receptor-beta (PDGFR-β), and low-density lipoprotein receptor (LDLR)—have been used to target therapeutic agents to activated HSCs for the treatment of liver fibrosis [19]. IGF2R, also known as mannose 6-phosphate receptor (M6PR), is a multifunctional receptor that regulates the bioavailability of insulin-like growth factor 2, activates the transforming growth factor-β (TGF-β), and transports M6P-tagged lysosomal proteins/enzymes. Chronic liver injury increases the expression of IGF2R on activated HSCs, making it a specific molecular marker of activated HSCs in the fibrotic liver [20]. We have previously discovered an IGF2R-specific peptide (peptide-431) using phage display technology, which can be used as a targeting ligand to deliver cargos to activated HSCs [21]. Dimerization of the peptide-431 further increases its binding affinity to human hepatic stellate cells LX-2 by about nine-fold, making it a potential targeting ligand for HSC-specific drug delivery.

In this study, we developed a cholesteryl peptide-based *Pcbp2* siRNA nanocomplex that is easy to fabricate. The surface of the nanocomplex was modified with a dimeric IGF2R peptide as a targeting ligand. We compared the use of dimeric IGF2R peptides with various glycine spacer and glutamate residues to increase the specific delivery of the siRNA nanocomplexes to activated HSCs. The dimeric IGF2R peptide ligand-modified siRNA nanocomplex with the highest cellular uptake and the most efficient silencing activity was evaluated for its biodistribution in rats with CCl_4_-induced liver fibrosis.

## 2. Materials and Methods

### 2.1. Materials

*Pcbp2* siRNA (sense strand: 5′-GUCAGUGUGGCUCUCUUAUdTdT-3′) and scrambled siRNA were ordered from Invitrogen (Carlsbad, CA, USA) and Genepharm (Shanghai, China), respectively. Cholesterol-modified peptide (Cholesterol-CHHHKKHHHKK) was ordered from United BioSystems Inc. (Herndon, VA, USA). Lipofectamine™ *RNAiMAX*, cell culture media, and all chemical reagents were purchased from Fisher Scientific (Pittsburgh, PA, USA).

### 2.2. Synthesis of the Dimeric IGF2R Peptide Ligands

The IGF2R peptide VHWDFRQWWQPS was discovered using phage display technology [21]. Dimeric IGF2R peptide ligands were synthesized using a PurePep Chorus peptide synthesizer (Gyros Protein Technologies, Tucson, AZ, USA). The dimeric IGF2R peptide ligands with various glycine spacers and glutamate residues were synthesized using solid phase peptide synthesis. The synthesized peptide ligands were purified with HPLC and their molecular weights were analyzed by ultra-high performance liquid chromatography–mass spectrometer (UPLC-MS).

### 2.3. Fabrication and Characterization of the Nanocomplex

The dimeric IGF2R peptide-modified cholesteryl peptide/siRNA nanocomplex was prepared by mixing siRNA and cholesteryl peptide in a one-pot, two-step procedure using manual pipetting. The siRNA and cholesteryl peptide were dissolved in diethylpyrocarbonate (DEPC)-treated water, respectively. Equal volumes of 20 µM siRNA and 800 µM cholesteryl peptide solution were vigorously mixed using a pipette to form the nanocomplex. The nanocomplex in N/P ratio 10:1 was stabilized at room temperature for 30 min, and 2 µL of 200 µM dimeric IGF2R peptide ligands was mixed with 10 µL of the nanocomplex and incubated for an additional 30 min at room temperature to form the final nanocomplex. The particle size and zeta potential of the dimeric IGF2R peptide-modified nanocomplex were analyzed using a Malvern Zetasizer Nano-ZS (Malvern Instruments, MA, USA). The critical micelle concentration (CMC) of the cholesteryl peptide was determined using pyrene as a fluorescent probe (Ex = 330 nm, Em (I1) = 372 nm, and Em (I2) = 392 nm) as previously described [16]. The morphology of the nanocomplex was evaluated using transmission electron microscopy (TEM) (Philips, Hamburg, Germany).

### 2.4. siRNA Entrapment and Serum Stability of the Nanocomplex

The siRNA entrapment efficiency and serum stability of the dimeric IGF2R peptide-modified nanocomplex were examined using an agarose gel retardation assay. Free siRNA and the nanocomplex were both loaded onto a 1% agarose gel prepared with GelRed^TM^ (Biotium, CA, USA) in TBE buffer. The unentrapped or released siRNA was examined by gel electrophoresis at 80 V for 1 h.

The serum stability of the IGF2R peptide-modified cholesteryl peptide/siRNA nanocomplexes was assessed by incubation with 50% rat serum at 37 °C and sampled at various time intervals. The collected nanocomplex was then incubated with an equal volume of 40 µM heparin and 10 mM glutathione for 10 min on ice to dissociate the encapsulated siRNA from the nanocomplex. The remaining siRNA was analyzed by electrophoresis in a 1% agarose gel and visualized with GelRed^TM^.

### 2.5. Cell Culture

The rat hepatic stellate cell line (HSC-T6) was kindly provided by Dr. Scott L. Friedman (Mount Sinai School of Medicine, New York University). HSC-T6 was cultured in Dulbecco’s Modified Eagle Medium (DMEM) (Corning, NY, USA) containing 10% fetal bovine serum (FBS) (Biowest, MO, USA), 100 units/mL penicillin, and 100 µg/mL streptomycin in a humidified atmosphere containing 5% CO_2_ at 37 °C.

### 2.6. Cytotoxicity of the Nanocomplex

Cytotoxicity of the cholesteryl peptide nanocomplex was compared to Lipofectamine™ RNAiMAX. Briefly, HSCs-T6 cells were seeded in 96-well plates (5000 cells/well) and incubated with the scrambled siRNA condensed with Lipofectamine™ RNAiMAX or cholesteryl peptide at 50, 100, and 200 nM siRNA at 37 °C for 24 and 48 h, then evaluated by CellTiter-Glo Kit (Promega, Madison, WI). Meanwhile, the cytotoxicity of dimeric IGF2R peptide ligand-modified cholesteryl peptide siRNA nanocomplexes at 100 nM siRNA were evaluated in HSCs-T6 cells for 24 h. Untreated cells served as the negative control, and the cells treated with 1% Triton X-100 served as the positive control.

### 2.7. Cellular Uptake of the Nanocomplex

Cellular uptake of the dimeric IGF2R-modified nanocomplex was evaluated in HSC-T6 cells as described [13]. In brief, HSC-T6 cells (1 × 10^5^ cells/well) were transfected with a Cy5-labeled siRNA-loaded nanocomplex at 37 °C for 1, 2, and 4 h. The cells were then washed with 1 mg/mL heparin-containing DPBS (Corning, NY, USA) for 5 min to minimize nonspecific interactions [13]. After trypsinization, the cells were washed and resuspended in DPBS, and the intensity of Cy5-labeled siRNA inside the cells was analyzed using a BD FACS II flow cytometer (BD instruments, Franklin Lakes, NJ, USA).

Fluorescent microscopy was used to validate the flow cytometry results. HSC-T6 cells were incubated with a Cy5-labeled siRNA-loaded nanocomplex for 2 and 4 h, washed with 1 mg/mL heparin-containing DPBS for 5 min, and then stained with LysoTracker Red DND-99 (Invitrogen, Carlsbad, CA, USA). The cells were fixed with 10% formalin and treated with Antifade Mounting Medium with DAPI (Vector Laboratories, Burlingame, CA, USA) before being examined under a fluorescent microscope (Keyence High Content Microscope, Osaka, Japan).

### 2.8. Silencing Activity of the Nanocomplex

The silencing activity of the dimeric IGF2R-modified nanocomplex with a N/P ratio of 10:1 was evaluated by the *Pcbp2* mRNA expression levels in HSC-T6 cells using the transfection method. Briefly, HSC-T6 cells (1 × 10^5^ cells/well) in a 12-well plate were transfected with scrambled or *Pcbp2* siRNA-loaded nanocomplexes at a final concentration of 100 nM siRNA for 4 and 24 h. After 4 h of incubation, the cells were washed with DPBS twice and incubated with fresh Opti-MEM for an additional 20 h. All the cells were harvested at 24 h post-transfection. The total RNA was isolated with TRIzol™ Reagent (Invitrogen, Carlsbad, CA, USA), and analyzed with a real-time PCR detection system (Bio-Rad, Hercules, CA, USA) using the iTaq™ Universal SYBR^®^ Green One-Step Kit according to the manufacturer’s protocol. The silencing activity of PCBP2 at the mRNA level was calculated using the comparative cycle threshold method as reported [22], with *18S* as the reference.

### 2.9. In Vivo Biodistribution Study

The biodistribution study was performed in a rat model of liver fibrosis as described previously [13]. The animal protocol (protocol number: 1110) was approved by the University of Missouri-Kansas City (UMKC) Institutional Animal Care and Use Committee (IACUC). Six male Sprague Dawley rats were intraperitoneally injected with a mixture of CCl_4_ and olive oil (1:1, *v*/*v*) at a dose of 1 mL/kg CCl_4_ twice a week for 4 weeks. The rats were then divided into two groups and intravenously administered with a free Cy5-labeled *Pcbp2* siRNA and a dimeric IGF2R-modified Cy5-labeled siRNA-loaded nanocomplex at a dose of 1 mg/kg siRNA. After 2 h, the rats were euthanized, and their organs, including the liver, lungs, spleen, kidneys, heart, and blood, were harvested for image analysis using a Bruker MS FX PRO In Vivo Imaging System (Billerica, MA, USA). The fluorescence intensity in the region of interest (ROI) was determined using Bruker molecular imaging software.

### 2.10. Statistical Analysis

All data were presented as means ± standard deviation. The statistical analysis was performed using Excel and GraphPad Prism 6.0. The difference in significance was determined using a two-tailed *t*-test or a one-way analysis of variance (ANOVA) followed by Tukey’s multiple comparison test. *p* < 0.05 will be considered statistically significant.

## 3. Results

Previously, we incorporated biotin-conjugated IGF2R peptide ligand into neutravidin-based nanocomplexes for specific drug delivery to HSCs. However, the process of fabricating these targeted neutravidin-based nanocomplexes was complicated and involved biotinylation modification as well as the additional protamine to stabilize the siRNA nanocomplex [13,14]. Therefore, we developed a peptide-based siRNA nanocomplex that was easy to fabricate.

### 3.1. Fabrication and Characterization of the Nanocomplex

**Figure 1:**
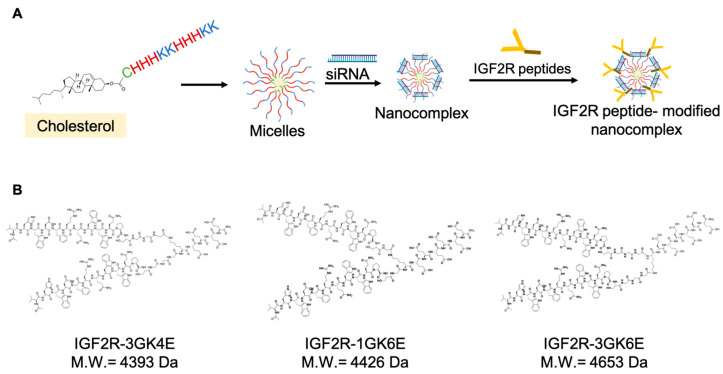
Schematic diagrams of the siRNA nanocomplex. (**A**) The fabrication schemes of dimeric IGF2R peptide-modified siRNA nanocomplex. (**B**) The structure of dimeric IGF2R-3GK4E peptide, IGF2R-1GK6E peptide, and IGF2R-3GK6E peptide. Cholesteryl peptide and *Pcbp2* siRNA were mixed in N/P ratio 10:1 at room temperature, followed by condensation with IGF2R peptide ligands to form the final nanocomplex.

**Figure 2:**
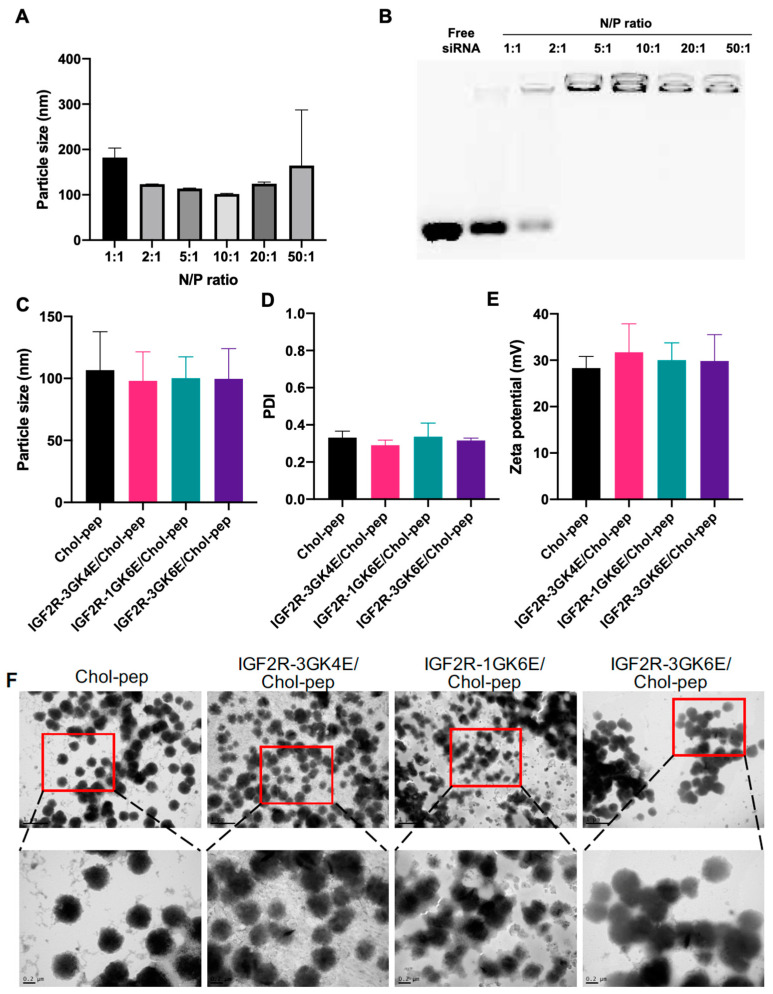
Characterization of the nanocomplex. (**A**) Particle size and (**B**) gel retardation assay of the cholesteryl peptide nanocomplexes at different N/P ratios. (**C**) Particle size, (**D**) PDI, (**E**) Zeta potential, (**F**) TEM images of the dimeric IGF2R peptide-modified nanocomplexes.

The nanocomplex was fabricated as illustrated in Figure 1A. Cholesteryl peptides and *Pcbp2* siRNA were mixed in various N/P ratios to form the nanocomplex (Figure 2A). As shown in Figure 2B, the nanocomplexes mixed in N/P ratios from 5:1 to 50:1 were able to completely encapsulate the siRNA. Based on the results of particle size, polydispersity index (PDI), and micelle stability, the nanocomplex mixed in an N/P ratio 10:1 was chosen for further surface modification. The dimeric IGF2R peptide ligands coupled with glutamates were mixed with the nanocomplex to form the formulation through electrostatic interaction. The particle size (Figure 2C) of the unmodified nanocomplex, dimeric IGF2R-3GK4E-modified nanocomplex, dimeric IGF2R-1GK6E-modified nanocomplex, and dimeric IGF2R-3GK6E-modified nanocomplex were 107, 98, 100, and 99 nm, respectively, and the PDI values (Figure 2D) were 0.331, 0.290, 0.336, and 0.316, respectively. The zeta potential of the nanocomplexes remained positively charged after surface modification, at approximately +30 mV (Figure 2E). The main characteristics of the IGF2R peptide-modified nanocomplex did not manifest significant differences from the unmodified nanocomplex. The morphologies of the nanocomplexes were examined with TEM as shown in Figure 2F. TEM images revealed that both the unmodified and the IGF2R peptide-modified nanocomplexes were mainly spherical nanoparticles.

### 3.2. Serum Stability and Cytotoxicity of the Nanocomplex

**Figure 3:**
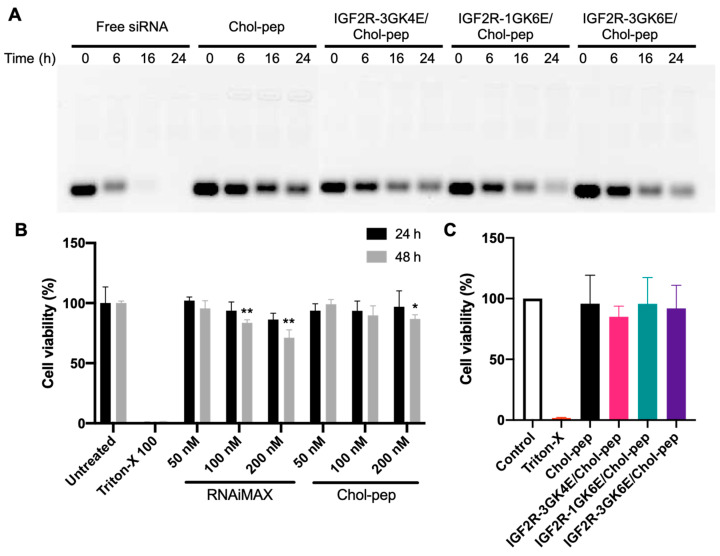
Serum stability and cytotoxicity of the nanocomplex. (**A**) Serum stability of siRNA nanocomplexes in 50% rat serum for 0, 6, 12, and 24 h. (**B**) Cytotoxicity of the cholesteryl peptide nanocomplexes and Lipofectamine™ RNAiMAX at 50, 100, 200 nM siRNA. (**C**) Cytotoxicity of the IGF2R peptide-modified nanocomplexes at 100 nM siRNA. Results are presented as the mean ± SD (*n* = 3) (* *p* < 0.05; ** *p* < 0.01).

One of the challenges in siRNA delivery is its rapid nuclease degradation and clearance in the body, which limits its transport to the target site. To address this issue, nanocomplex formulations have been developed to prolong the half-life of siRNA. The nanocomplex formulations were incubated in 50% rat serum for 0, 6, 16, and 24 h to evaluate their ability to protect siRNA from degradation. As shown in Figure 3A, free siRNA was degraded in the serum after 6 h and was nearly undetectable after 16 h. Heparin and glutathione were used to dissociate the entrapped siRNA from the nanocomplexes. The results showed that the nanocomplex formulations were able to effectively protect siRNA from degradation in the serum for up to 24 h. In addition, the cholesteryl peptide/siRNA nanocomplex demonstrated less cytotoxicity than Lipofectamine™ RNAiMAX at high concentrations in HSC-T6 cells (Figure 3B). There was no significant cytotoxicity observed in HSC-T6 cells treated with the dimeric IGF2R peptide ligand-modified nanocomplex at a concentration of 100 nM siRNA (Figure 3C).

### 3.3. Cellular Uptake of the Nanocomplex

**Figure 4:**
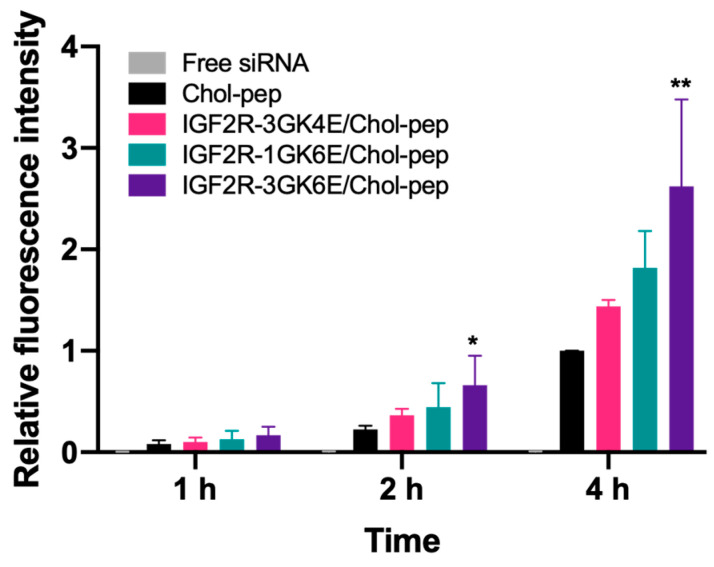
Quantitative cellular uptake of IGF2R peptide-modified nanocomplex in HSC-T6 cells. The cells were incubated with a nanocomplex loaded with Cy5-labeled *Pcbp2* siRNA for 1, 2, and 4 h, followed by flow cytometry analysis. Results are presented as the mean ± SD (*n* = 3) (* *p* < 0.05; ** *p* < 0.01).

**Figure 5:**
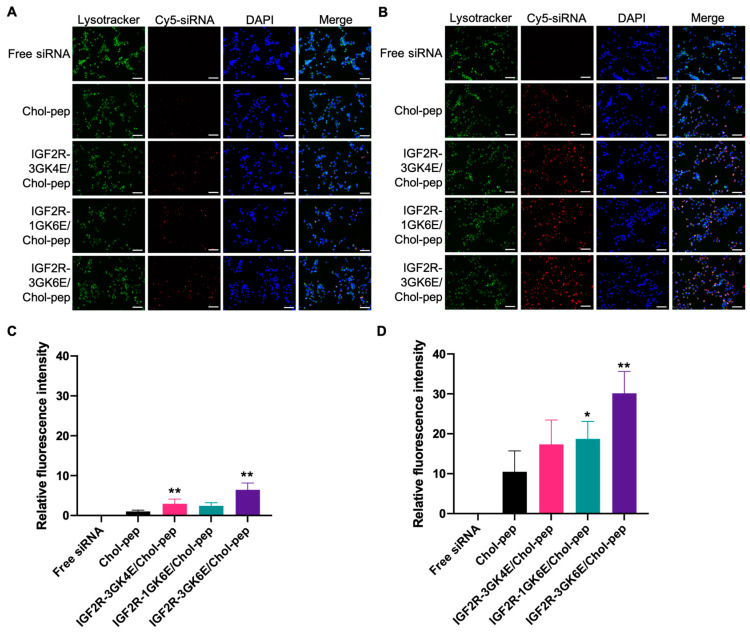
Cellular uptake study of a IGF2R peptide-modified nanocomplex in HSC-T6 cells using fluorescent microscopy. HSC-T6 cells were examined after incubation with free Cy5-labeled siRNA, Cy5-labeled siRNA nanocomplex, and IGF2R peptide-modified Cy5-labeled siRNA nanocomplex for 2 h (**A**,**C**) and 4 h (**B**,**D**). (**A**,**B**) Representative fluorescence images (scale bar = 100 µm). (**C**,**D**) Quantitative fluorescence analysis by ImageJ. Results are presented as the mean ± SD (*n* = 6 images; * *p* < 0.05; ** *p* < 0.01).

Cy5-labeled *Pcbp2* siRNA was used in the study to evaluate the cellular uptake of the IGF2R peptide-modified nanocomplexes in HSC-T6 cells (Figure 4). As shown in the results, the cellular uptake of free siRNA was limited. Compared to free siRNA, the unmodified nanocomplex significantly increased fluorescence intensity nearly 17-fold, 41-fold, and 152-fold at 1, 2, and 4 h post-incubation, respectively. The use of a cholesteryl peptide to form the nanocomplex facilitated the cellular uptake of Cy5-labeled *Pcbp2* siRNA in a time-dependent manner. Additionally, the IGF2R peptide-modified cholesteryl peptide nanocomplex significantly increased fluorescence intensity in transfected cells, which was consistent with our previous finding that the IGF2R peptide-modified neutravidin-based nanocomplex showed high cellular uptake in activated LX-2 cells and HSC-T6 cells [13]. In particular, the dimeric IGF2R-3GK6E peptide-modified nanocomplex showed the highest fluorescence intensity, which was approximately 2.7 times higher than the fluorescence intensity of the unmodified nanocomplex after incubation for 4 h (Figure 4). Fluorescent microscopy was also used to examine the cellular uptake of the nanocomplexes. In accordance with the flow cytometry results, the images revealed that the dimeric IGF2R peptide-modified nanocomplex had the highest fluorescent intensity (Figure 5). These results indicate that the dimeric IGF2R peptide-modified nanocomplex can effectively deliver siRNA into HSC-T6 cells.

### 3.4. Silencing Activity of the Nanocomplex

**Figure 6:**
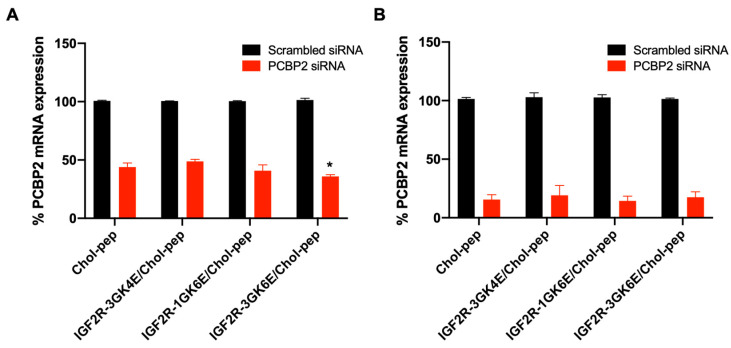
Silencing activity of the *Pcbp2* siRNA in HSC-T6 cells. mRNA expression of the *Pcbp2* gene in HSC-T6 cells after incubation with the *Pcbp2* siRNA nanocomplex for (**A**) 4 h and (**B**) 24 h. Results are presented as the mean ± SD (*n* = 3) (* *p* < 0.05).

The cholesteryl peptide/*Pcbp2* siRNA nanocomplex efficiently silenced approximately 80–85% of *Pcbp2* mRNA expression in activated HSC-T6 cells after transfection for 24 h (Figure 6B). This demonstrated excellent silencing activities, which is consistent with the literature on cholesteryl peptide-based nanocomplexes applied in different cell lines [16,17,18]. Based on the cellular uptake results (Figure 4 and Figure 5), the silencing activity of the nanocomplex was further evaluated for 4-h transfection in activated HSC-T6 cells. The IGF2R-3GK6E peptide-modified nanocomplex demonstrated a higher silencing activity in activated HSC-T6 cells compared to the bare nanocomplex, indicating that the IGF2R peptide facilitated specific targeting and uptake of the nanocomplex (Figure 6A).

### 3.5. In Vivo Biodistribution Study

**Figure 7:**
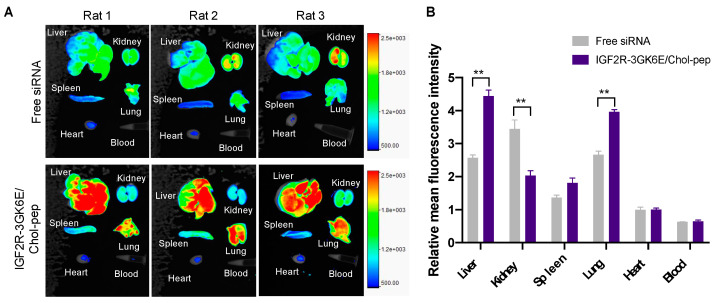
Biodistribution of the nanocomplexes in rats with CCl_4_-induced liver fibrosis. The rats were injected with free Cy5-labeled siRNA and IGF2R-3GK6E-modified Cy5-labeled siRNA nanocomplex at 1 mg/kg siRNA via tail vein, respectively. The rats were euthanized for 2 h post-injection, and the organs including the liver, kidneys, spleen, lungs, heart, and blood were harvested for fluorescence analysis. (**A**) Fluorescence images of the major organs. The region of interest (ROI) for each organ was determined by the Bruker molecular imaging software. (**B**) Relative mean fluorescence intensities of the major organs. Results are presented as the mean ± SEM (*n* = 3). (** *p* < 0.01).

Based on the results of the cellular uptake of the dimeric IGF2R peptide-modified nanocomplex in HSC-T6 cells, we next examined the biodistribution of the nanocomplexes in rats with CCl_4_-induced liver fibrosis (Figure 7). As expected, after two hours post intravenous injection, free Cy5-labeled *Pcbp2* siRNA showed a lower fluorescence intensity in most organs and higher kidney accumulation due to rapid clearance. In contrast, the IGF2R peptide-modified nanocomplex significantly increased the accumulation of Cy5-labeled *Pcbp2* siRNA in the fibrotic liver. The results indicated that the IGF2R peptide-modified nanocomplex is a promising platform for effectively protecting and specifically delivering antifibrotic siRNA to the fibrotic liver in vivo.

## 4. Discussion

In our previous studies, the IGF2R peptide-modified biotin-neutravidin-based siRNA nanocomplex showed excellent performance in vitro and in vivo [13,14]. However, protein-based therapeutics tend to induce undesired immune responses, which can result in life-threatening side effects [15]. To reduce the risk of high immunogenicity, small peptides with short sequences of amino acids can be an alternative biomaterial for nanoscale delivery systems. Moreover, polyaminoacids/polypeptides provide adjustable and flexible compositions that can be tailored to meet the requirements of various drug-delivery applications [23].

We previously developed a small peptide-based delivery system that primarily consists of lysine and histidine residues [16]. Cholesterol was conjugated to the N-terminus of the peptides to form an amphiphilic molecule that could self-assemble into a micelle-like structure when the concentration of cholesteryl peptides is above its CMC (16 µM) (Figure 1A). The side chains of lysine and histidine are positively charged at physiological pH, which can promote the condensation of negatively charged siRNA through electrostatic interactions. The cholesteryl peptide nanocomplex can be fabricated in N/P ratios from 5:1 to 50:1 to completely encapsulate *Pcbp2* siRNA, which showed a high encapsulation efficiency (Figure 2B). The siRNA nanocomplex with an N/P ratio of 10:1 showed the smallest particle size (101.8 nm) compared to nanocomplexes with other N/P ratios (Figure 2A). Additionally, the incorporation of cysteine into cholesterol and peptides can effectively improve micelle stability and transfection efficiency [17].

In many studies, delivery materials for siRNA such as cationic lipid, polyethyleneimine (PEI), and poly-L-lysine may cause potential toxicity due to their high positive charge [24]. The toxicity of cationic gene delivery nanoparticles can be reduced using the simple and effective approach of coating the nanoparticles with polyglutamate [25]. Many studies have reported that polyglutamate can effectively reduce the cytotoxicity of cationic cell-penetrating peptides through covalent or non-covalent interaction [26,27]. The length and amount of polyglutamate can be adjusted to prevent toxicity while maintaining cellular uptake efficiency [28]. Furthermore, negatively charged polyglutamate can be utilized for structure stabilization and surface modification. Studies reported that polyglutamate-grafted polyethylene glycol (PEG) can shield the surface of positively charged nanoparticles via electrostatic interaction to extend blood circulation time [29,30,31].

To effectively transport antifibrotic siRNA into HSCs, our previous findings suggested that a nanocomplex modified with the dimeric IGF2R peptide ligand significantly increased in vitro cellular uptake in HSCs and specifically delivered siRNA to the fibrotic liver as opposed to a nanocomplex modified with vitamin A and cholesterol [13]. In this study, we designed peptide-based ligands that conjugated the IGF2R peptide with a short spacer and glutamate residues to modify the surface of the cholesteryl peptide/*Pcbp2* siRNA nanocomplex (Figure 1). The incorporation of glutamate residues into the C-terminus of the IGF2R peptides provided a negative charge at physiological pH, which facilitated the IGF2R ligands to condense on the surface of the positively charged nanocomplex. As shown in Figure 4 and Figure 5, six glutamate residues showed higher cellular uptake than four glutamate residues. These results suggest that an increased number of glutamate residues may enhance interactions with the nanocomplex, allowing more ligands to stably condense on the nanocomplex. Furthermore, the short spacer, comprised of glycine, played an important role in separating the IGF2R peptide ligand from the interface [32]. These cellular uptake data suggest that the IGF2R peptide ligand conjugated with three glycine residues as a spacer may be more effective in binding to the IGF2R than a single glycine. Overall, a nanocomplex modified with IGF2R peptide ligands conjugated with three glycine residues and six glutamate residues (3GK6E) demonstrated the highest cellular uptake in HSC-T6 cells.

In accordance with the literature, the cholesteryl peptide delivery system showed the potent silencing activity of *Pcbp2* siRNA in HSC-T6 cells (Figure 6). As reported in the cellular uptake study, a nanocomplex modified with IGF2R-3GK6E peptide ligand significantly downregulated mRNA expression of the *Pcbp2* gene after 4 h of incubation compared to the bare nanocomplex, indicating a high transfection efficiency. This result was attributed to the overexpression of IGF2R on the cell membrane of activated HSC-T6 cells. The upregulated expression of IGF2R facilitated approximately three times the endocytosis and mediated intracellular transportation [33]. Therefore, the in vitro cellular uptake and silencing activity studies suggest that IGF2R-3GK6E peptide-modified nanocomplex can increase HSCs-specific targeting and siRNA delivery efficiency of the nanocomplex. An in vivo biodistribution study was performed in a rat model with CCl_4_-induced liver fibrosis to evaluate the uptake of IGF2R peptide-modified nanocomplex in the fibrotic liver (Figure 7). Nanoparticles with positively charged surfaces tend to accumulate in the liver, lung, and spleen [34]. Chol-pep nanocomplex effectively prevented the siRNA from nuclease degradation during blood circulation [17]. In accordance with the literature [13,14], the IGF2R-3GK6E peptide-modified nanocomplex demonstrated a significant uptake in the liver, prolonged the half-life of siRNA, and reduced renal clearance, making it a promising siRNA delivery system for the treatment of liver fibrosis.

In summary, we developed a targeted peptide-based siRNA delivery system for HSCs. The nanocomplex significantly improved the serum stability and silencing activity of siRNA. The incorporation of glycine and glutamate residues into the dimeric IGF2R peptide ligand allowed for the easy modification of the Chol-pep/siRNA nanocomplex through electrostatic interactions. The IGF2R peptide modification successfully increased in vitro transfection efficiency and the cellular uptake of the nanocomplex in activated HSC-T6 cells, as well as in vivo accumulation in the fibrotic liver. Therefore, the IGF2R peptide-modified nanocomplex has the potential to be a useful delivery system to specifically deliver therapeutic siRNAs to activated HSCs in the fibrotic liver.

## Data Availability

The data that support the findings of this study are available from the corresponding author, K.C., upon reasonable request.
